# Robustness and efficiency of international pesticide trade networks subject to link removal strategies

**DOI:** 10.1038/s41598-022-21777-1

**Published:** 2022-11-16

**Authors:** Wen-Jie Xie, Jian-An Li, Na Wei, Li Wang, Wei-Xing Zhou

**Affiliations:** 1 School of Business, East China University of Science and Technology, Shanghai, 200237 China; 2 Research Center for Econophysics, East China University of Science and Technology, Shanghai, 200237 China; 3 School of Physics, East China University of Science and Technology, Shanghai, 200237 China; 4 School of Mathematics, East China University of Science and Technology, Shanghai, 200237 China

**Keywords:** Applied physics, Statistical physics, thermodynamics and nonlinear dynamics

## Abstract

The international pesticide trade network (iPTN) is a key factor affecting global food production and food security. The trade relationship is a key component in iPTNs. In a complex international trade environment, we model the impacts of uncertain factors such as trade wars, economic blockades and local wars, as removing vital relationships in the trade network. There are many complex network studies on node centrality, but few on link centrality or link importance. We propose a new method for computing network link centrality. The main innovation of the method is in converting the original network into a dual graph, the nodes in the dual graph corresponding to the links of the original network. Through the dual graph, the node centrality indicators can measure the centrality of the links in the original network. We verify the effectiveness of the network link centrality indicator based on the dual graph in the iPTN, analyze the relationship between the existing network link centrality indicators and the indicator proposed in this paper, and compare their differences. It is found that the trade relationships with larger indicators (hub, outcloseness, outdegree) based on the dual graph have a greater impact on network efficiency than those based on the original pesticide trade networks.

## Introduction

Complex systems are ubiquitous, and their complexity lies in the large number of individuals and particularly complex relationships in the system. In general, a large number of relationships between individuals constitute the main structural and evolutionary features of complex systems. Complex network is an important tool and method to study complex systems. In the real world, the social environment and trade environment are extremely complex. The local wars, COVID-19, and trade wars have all directly affected the international situation and trade environment. In the meantime, many uncertain factors, such as trade wars, economic sanctions and trade blockades, have affected the development of international pesticide trade relations. These factors can be modeled as the removal of trade links in the trade network. In order to prevent the breakdown of some important trade relationships in advance, it is necessary to identify some important trade relationships, that is, important network links. The removal of these links will affect network functions such as network efficiency and network connectivity, thus making a large impact.

The index of vital links in networks can be measured by the centrality, importance and influence of network links. The centrality of network nodes has been studied by many researchers and widely applied in different fields^1–5^. In collaborative networks of scientists, node centrality can be used to identify influential scientists^6,7^. In financial networks, researchers analyze, for instance, the relationship between the CEO network centrality and merger performance^8^, the boardroom centrality and firm performance^9^, the network centrality and delegated investment performance^10^, and the trade network centrality and currency risk premia^11^. In sports networks, the player centrality in the passing network of world cup soccer teams can reveal many interesting phenomena^12–14^.

Network analysis has an important position in socioeconomic system^15–17^, especially in financial or economical systems, and research on the importance and centrality of individuals has been the focus of attention^18,19^. In the socioeconomic system the economic agent is too big to fail, too connected to fail, and too central to fail^20,21^. The importance and centrality of network nodes have always provided important decision-making reference indicators for decision-makers^22^. Network links also play a key role in complex systems^2,11^, but there are few methods and literature for measuring and analyzing network link centrality.

The main idea of this paper comes from the literature on network link control^23^. Nepusz et al. introduced dual graph theory and analyzed the controllability of network links. Therefore, we also introduce the importance and centrality of links in the dual graph. The importance of the nodes in the dual graph is used as an indicator for the importance of the links in the original network. The influence of relationships on network structure and dynamic characteristics provides a new calculation method and indicator for identifying important trade relationships and vital links in socioeconomic network^24^.

In the international trade system, trade relationships have always been the focus of research^25–27^. Identifying important trade relationships and key trade behaviors has always been a hot topic of research. In this paper, the importance indicator of network links based on dual graph is used in the international pesticide trade network (iPTN) to analyze the importance of the trade relationships of different pesticide products and the impact on the stability of network structure^28,29^. The trade network of agricultural products directly affects international food security^30^ and the basic functioning of socioeconomic activities^31–33^. The international trade network has attracted the attention of researchers from different disciplines around the world^34–38^.

The robustness^39–43^ and efficiency^41^ of the network has always been a hot topic in network science. The important links will directly affect the dynamic characteristics of the network, which is of great significance to the robustness and efficiency of network structure^42^. When the network is subjected to random shocks or targeted attacks, it will affect the network stability. The types of shocks or the importance of the impacted links vary, and the degree of network impact varies as well^44,45^. We will verify the effectiveness of the method proposed in this paper by analyzing the influence of vital links on the robustness and efficiency of networks^18,19^.

## Results

### Construction of international pesticide trade networks

**Figure 1:**
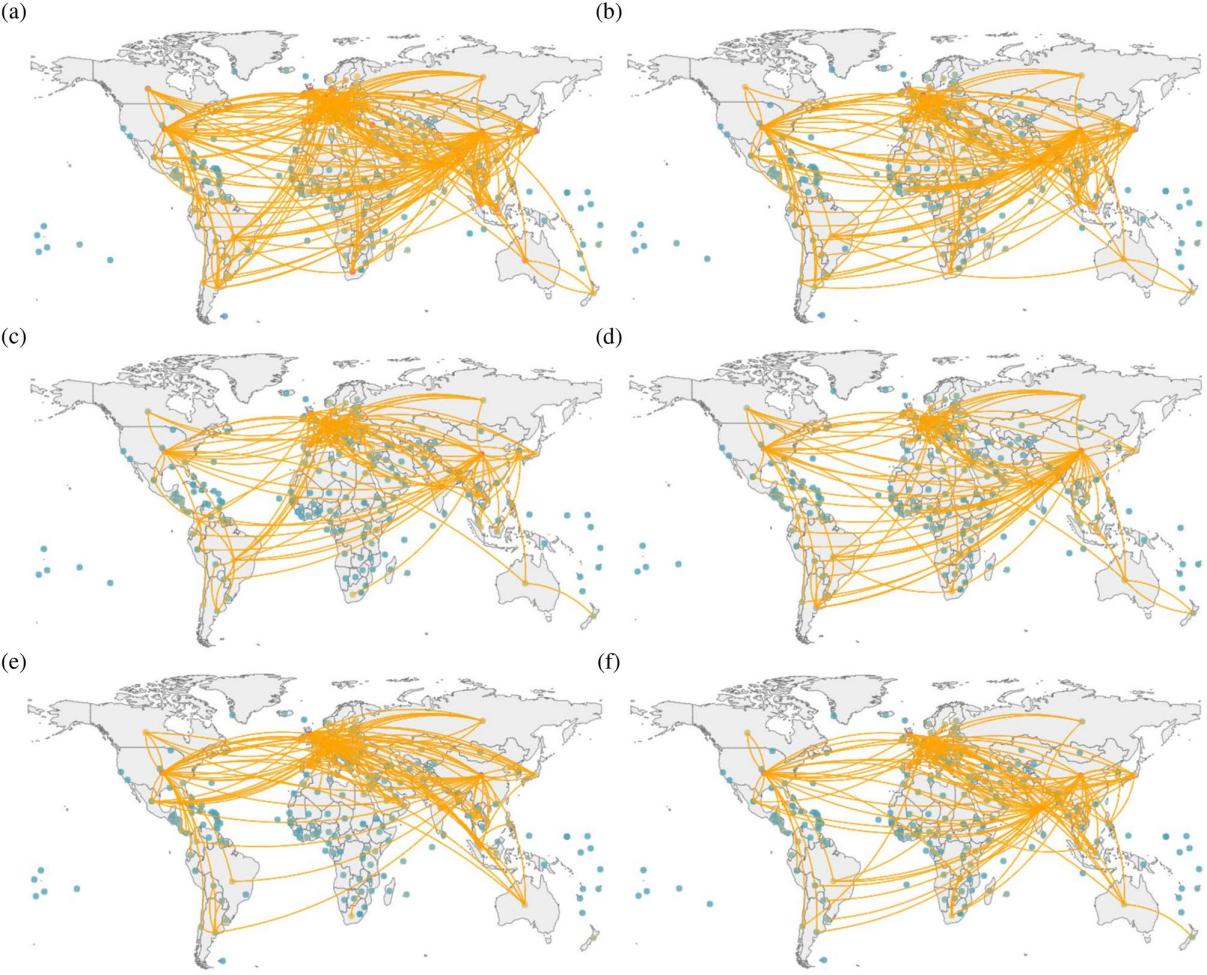
An example of the topology of the international pesticide trade network (iPTN) with part of links in 2018. The six plots correspond to the trade networks of pesticide products, including (**a**) aggregated, (**b**) insecticides, (**c**) fungicides, (**d**) herbicides, (**e**) disinfectants, and (**f**) rodenticides and other similar products.

We use the international pesticide trade data published on the official website of UN Comtrade from 2007 to 2018. The data includes exporting economies, importing economies, and trade volumes. $\mathbf {V}(t)=[v_{ij}(t)]$ represents the pesticide trade volume of economy *i* exported to economy *j* in year *t*. Figure 1a shows the aggregated trade network of 5 pesticide products, while Fig. 1b–f illustrate the pesticide trade networks for insecticides (380891), fungicides (380892), herbicides (380893), disinfectants (380894), and rodenticides and other similar products (380899)^46^. Each plot retains 5% of the trade relationships with the highest volume. The topology and spatial distribution of different pesticide trade networks in Fig. 1 have similar structures. But there are also differences in some local areas. The major reason is that they all need to be based on the industrial production conditions and supply chain conditions of the economy, although the production processes of different pesticides are different.

We can intuitively understand the spatial distribution of the iPTN in Fig. 1. The trade networks of different types of pesticide products share certain similarities. In addition, Europe, the United States, China, Brazil, Australia and other major agricultural economies occupy obvious prominent positions in the networks.

### Construction of dual graph of international pesticide trade networks

Based on the trade volume matrix $\mathbf {V}$, we calculate the adjacency matrix $\mathbf {A}=[a_{ij}]$ of the pesticide trade network between economies. The adjacency matrix element $a_{ij}=1$ represents the trade volume $v_{ij}>0$ between economy *i* and economy *j* and $a_{ij}=0$ otherwise. In order to analyze the links’ centrality in the network, we adopt the graph theory and convert the directed network $\mathbf {A}=[a_{ij}]$ into a dual graph, which is used to analyze the nature of the trade relationship of the original trade network.

**Figure 2:**
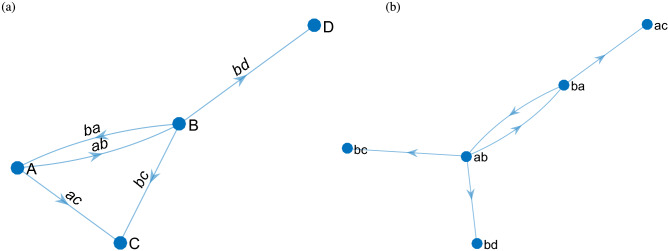
Example of building a dual graph. (**a**) Original graph. (**b**) Dual graph.

We denote the original directed network as $G=\langle V,E\rangle$, where $V=\{v_1,v_2,...,v_N \}$ represents the original network node set, and $E=\{e_1,e_2,...,e_M\}$ represents the link set. The dual graph of original network $G=\langle V,E\rangle$ can be expressed as $G^{*}=\langle V^{*},E^{*}\rangle$, where $V^{*}=\{e_1,e_2,..., e_M\}$ represents the set of nodes in the dual graph, which is the set of links in the original network. Each link in the original network corresponds to a node in the dual graph. When the original network is a directed graph, the link construction in the dual graph needs to preserve the property of the direction. Figure 2 is a simple example of converting the original graph to the dual graph. The original graph contains 4 nodes $\{A,B,C,D\}$ and 5 links $\{ab,ba,ac,bc,bd\}$. Therefore, there will be 5 nodes in the dual graph with $V^{*}=\{ab,ba,ac,bc,bd\}$. The key to the construction of dual graphs is to determine relational links. For example, based on whether a link exists between node *ab* and *bc*, it is necessary to consider whether the links in the original network corresponding to the two nodes can be connected. Since there is a directed path $\{a,b,c\}$ in *G*, we obtain a directed link from *ab* to *bc* in $G^*$. In contrast, there is no link connecting *ac* and *bc* in $G^*$ when there is no directed path in *G*, although the two links in *G* share a vertex.

### Centrality indicators for nodes in international pesticide trade networks

There are many node centrality indicators in network analysis. We select 9 node centrality indicators^47–49^: hubs, out-degree, in-degree, authorities, PageRank, incloseness, outcloseness, betweenness, and clustering. The degree of a node describes the number of links connected to it. For a directed network, the in-degree represents the number of links that point to a node, and the out-degree represents the number of points from a node to others. The closeness is the reciprocal of the sum of the distances of a node to all other nodes. If the node cannot reach any other nodes, the closeness is zero. In-closeness measures the ease of other nodes to reach this node. The higher the in-closeness of an economy in iPTN, the easier it is for other economies to reach this economy. Out-closeness measures the ease of a node to reach other nodes. The higher the out-closeness, the easier it is for an economy to reach other economies. Betweenness measures the frequency of occurrence of a node on the shortest path between two nodes in the network^50,51^. PageRank centrality is based on Google’s sorting algorithm^52^. The hubs and authorities centrality are two recursively defined centrality values. The clustering coefficient of a node measures the proportion of connections between the node’s neighbor nodes. A node’s hub score is the sum of the authority scores of all its successor nodes. Likewise, the authority score is the sum of the hub scores of all its predecessor nodes. The sum of all the hub scores is equal to 1, and the sum of all authority scores is equal to 1 as well.

### Centrality indicators for links in international pesticide trade networks

The above definitions are all based on the centrality measurement of nodes. This article is intended mainly to calculate the centrality of links through the measurement of node centrality on a dual graph. The nodes in the dual graph are the links in the original network. It is necessary to calculate the centrality of the links in the original network, as the importance and centrality of the nodes in the dual graph are calculated. In order to analyze the effectiveness of our method, we also adopt six existing methods to measure the importance and centrality of network links.

In a directed weighted network, the most direct and commonly used indicator to measure the importance of a link is its weight. In most applications, link weights cannot reflect the importance of trade relations of some small economies. Many importance indicators are defined based on network structure and contain network structure information. Edge betweenness (EB) is the most representative indicator of centrality for a link. Significance $\alpha _{ij}$ of link is based on the method of Serrano et al.^53^. CN is defined as the common neighbors of the two nodes corresponding to the link. Resource Allocation ($\mathrm{{RA}}$)^54^ is similar to Adamic/Adar ($\mathrm{{AA}}$)^55^. The details of each variable calculation are described in Section *Materials and methods*.

Thus far, nine indicators based on node centrality and six indicators based on link centrality have been defined. In order to make a simple comparison of the 15 indicators, we calculated the centrality values of nodes (trade relations) in the dual graph using the node centrality indicators, and also calculated the 6 link centrality indicators in the original network. for each trade relationship in the original trade network, 15 indicators for vital links can be obtained, among which the trade volume (weight) of the trade relationship is the most direct and commonly used indicator.

### Correlation between link centrality indicators

**Figure 3:**
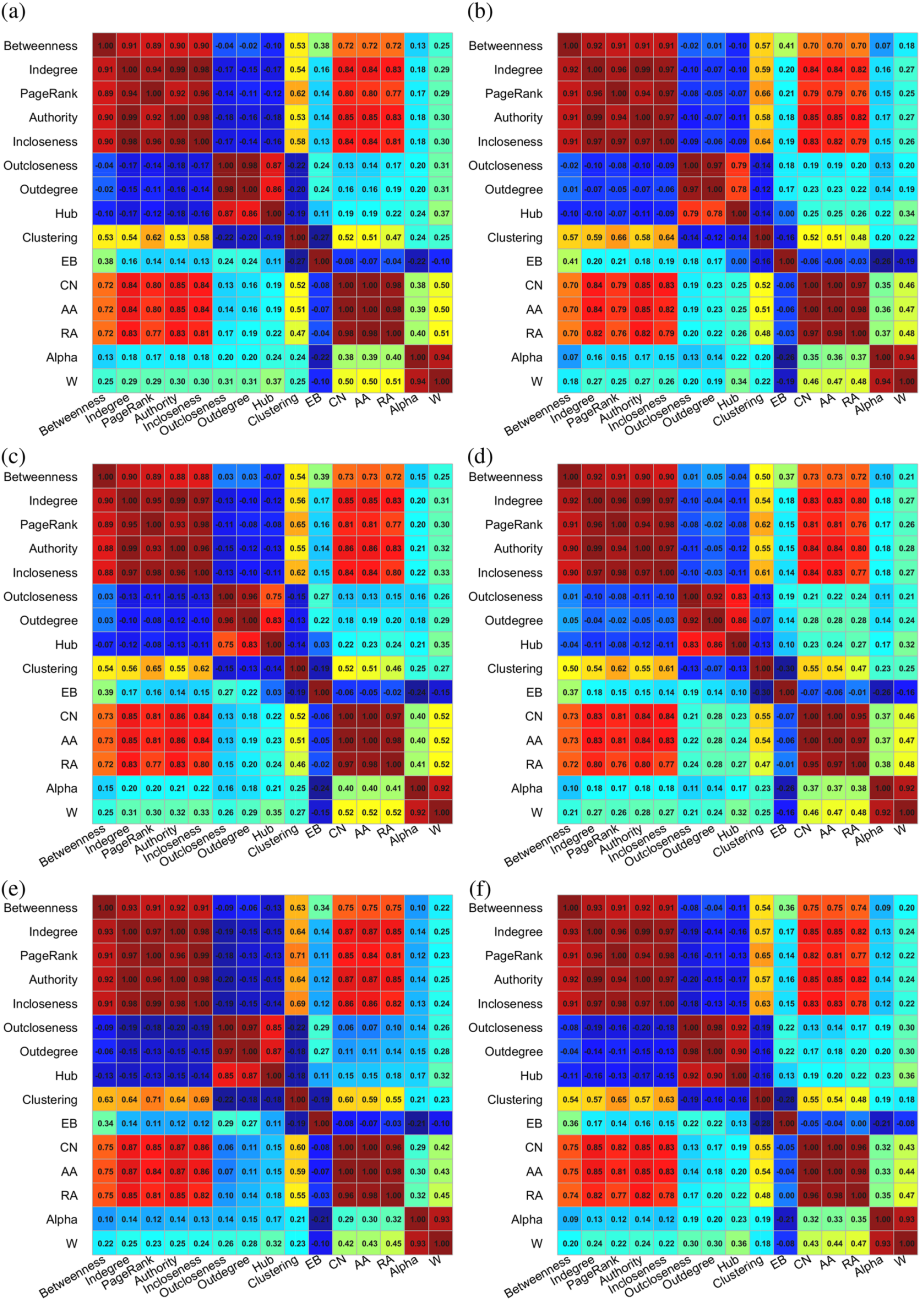
Spearman’s rank correlation coefficients between link centrality indicators in international pesticide trade networks in 2018. The six plots correspond to the trade networks of pesticide products, including (**a**) aggregated, (**b**) insecticides, (**c**) fungicides, (**d**) herbicides, (**e**) disinfectants, and (**f**) rodenticides and other similar products.

Figure 3 shows the correlation coefficients between the link centrality based on the network structure, and each plot corresponds to a type of pesticide trade network. Since the values of different indicators are quite different, Spearman’s rank correlation coefficient is used to describe the relationship between different indicators. The nine edge centrality indicators based on the node centrality of the dual graph can be divided into three categories: betweenness, in-degree, PageRank, authority and in-closeness; out-closeness, out-degree and hub; clustering.

It can be found from the correlation between the six basic edge centrality indicators that most of the importance indicators have positive correlation coefficients, but the correlation between the link betweenness (EB) is not obvious when the significance level is 0.05. The correlation coefficient between Alpha and weight is significantly high, as Alpha is defined based on weights. Compared with the link centrality metrics based on dual graphs, The link centrality metrics based on the original network structure have higher correlation coefficients with weights, while EB is an exception. The six edge centrality indicators based on the original network can also be divided into three categories, and the classification can be seen can be seen from the block in the lower right corner of the correlation coefficient matrix.

There is also a high correlation between the five indicators (betweenness, in-degree, PageRank, authority and in-closeness) based on node centrality and the three indicators (CN, AA, RA) based on the original network edge centrality. Obviously, indicators with higher correlations will have similar impacts on network stability and efficiency. This conclusion can be verified from the analysis of network robustness and efficiency in the following section.

### Influence of vital links on robustness of international pesticide trade networks

We analyze the importance of trade relations corresponding to different indicators. It should be pointed out that the importance of trade relations corresponds to the issues to be analyzed, such as the famous weak ties theory in network science. Weak links can also have an important position in the corresponding networks. Therefore, we choose network stability^56^ as the background problem to measure the link centrality with different indicators.

**Figure 4:**
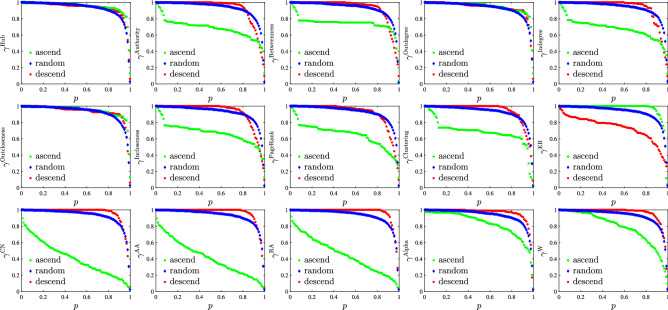
The relation between the proportion *p* of the deleted links and the proportion $\gamma ^\mathrm{{I}}$ of the number of economies in the largest component formed by the remaining network links after deleting the links according to the indicator *I*. In the legends, descend, ascend and random correspond to the cases of preferentially deleting the largest, smallest and random links with indicator *I*, respectively. Each plot corresponds to a link importance or centrality indicator.

Based on the indicator *I*, the trade relations in the network are deleted, and then the maximum connected subgraph formed by the remaining links is calculated as the largest component. There are three methods for removing trade relationships. The “descend” method is to firstly delete the trade relationship with the largest indicator *I*, the proportion of deleted links is *p*, and the remaining network of the largest component is obtained. The “ascend” method is to firstly delete less important links, that is, the trade relationship with the smallest indicator *I*. The “random” method is to randomly delete trade relations. Finally, we calculate the proportion $\gamma ^\mathrm{{I}}$ of the number of economies in the largest component,

1 $$\begin{aligned} \gamma ^\mathrm{{I}}(p)=\frac{N_{p}}{N}, \end{aligned}$$

the result is shown in Fig. 4.

It can be seen from Fig. 4 that the size of the giant component obtained by different link deletion methods has different variation patterns. When the trade relationship with the smallest indicator *I* first (ascend) is deleted, the size of the remaining network becomes smaller than that of the other two methods. This result is consistent with the previous theory about weak ties. the reason is that the links with the smallest indicator *I* represent some trade relations that play a strong linking role in the pesticide trade network. The smallest change of the largest component occurs in the case “descend” where the trade relations with the largest indicator *I* are firstly deleted. Those trade relations with the largest indicator *I* are the links with large trade volumes. These links have little effect on the connectivity of the network. The method of randomly removing the links of the trade network is somewhere in between. Here we randomly simulate 20 times, and then average the proportion $\gamma ^\mathrm{{I}}$ of the largest component in the case of “random”. In Fig. 4, when the proportion of deleted links reaches 80%, the proportion of economies in the largest component remains approximately 80%. This shows that most of the links in the trade network do not affect the connectivity of the network. The network has a certain resistance and can serve 80% of the world’s economies even when most of the network links fail.

**Figure 5:**
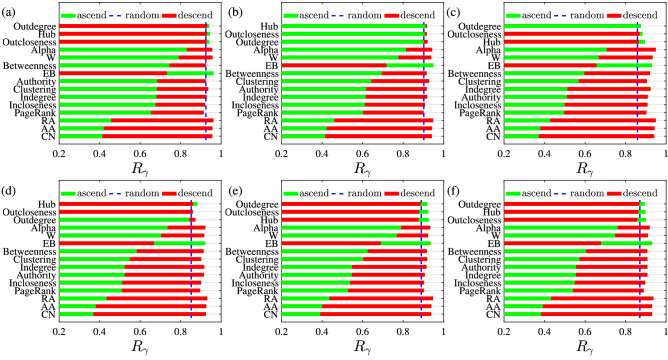
Comparison of the influence of different indicators on network robustness. In the legends, descend, ascend and random correspond to the cases of preferentially deleting the largest, smallest and random links of the indicator *I*, respectively. The six plots correspond to the trade networks of pesticide products, that is, (**a**) aggregated, (**b**) insecticides, (**c**) fungicides, (**d**) herbicides, (**e**) disinfectants, and (**f**) rodenticides and other similar products.

It can be seen from Fig. 4 that not all the links deleted in ascending order of the indicator will have a greater impact on connectivity (the proportion $\gamma ^\mathrm{{I}}$ of the number of economies in the largest component) than those links deleted in descending order. The most obvious in the figure is the plot corresponding to the EB indicator. The impact on network connectivity is significantly greater when the links are deleted in descending order of EB indicators than when the links are not deleted in descending order. This indicates that links with larger EB indicators have a greater impact on network connectivity. Other metrics are different, because based on weak ties theory, some relatively unimportant link connect some distant components. However, the definition of the EB indicator determines that the link with larger EB indicator are the key links connecting different modules or distant components. In Fig. 4, the difference in the influence of different indicators on network robustness corresponding to the indicators hub, outdegree and outcloseness are not obvious. In order to show in a clearer quantity way the differences between the indicators, the impact of each indicator on the robustness is given in Fig. 5.

In order to quantitatively compare the impact of different indicators on the robustness of network, the area under the curve in Fig. 4 is used to describe the corresponding impact degree of the indicator: the smaller the area, the greater the impact. The small size of the area represents an important role of the indicator in the stability of the network structure, and the calculation formula is

2 $$\begin{aligned} R_{\gamma }=\frac{1}{n+1}\sum _{k=0}^{n}\gamma \left( p_k\right) , \end{aligned}$$

where $p_k=k/n$ represents the proportion of deleted links, and *n* represents the number of simulated deletions^57^.

Each plot in Fig. 5 corresponds to a trade network. Figure 5a is the combined network of five international trade networks for pesticide products. The other five plots correspond to the trade networks of five pesticide products: insecticides (380891), fungicides (380892), herbicides (380893), disinfectants (380894), and rodenticides and other similar products (380899). The area under the curve in Fig. 4 corresponding to descending order is defined as $R_{\gamma }^\mathrm{{descend}}$, and the area corresponding to ascending order is recorded as $R_{\gamma }^\mathrm{{ascend}}$. We are more concerned with finding the metric that has the greatest impact on the robustness of network, regardless of the ordering rules for removing edges. Each indicator in Fig. 5 is sorted according to $\min {(R_{\gamma }^\mathrm{{ascend}},R_{\gamma }^\mathrm{{descend}})}$.

Different colors and markers correspond to different deletion rules. The importance indicators CN, AA and RA defined by the original network trade relationship play the most significant roles among all the indicators, and the importance indicators based on the dual graph also have significant effect, such as PageRank, Incloseness, Auth and Indegree. However, the link betweenness (EB) of the original network is not as good as the centrality indicator defined based on the dual graph. It can be found in Fig. 5 that the ranking of different indicators in the 6 networks is considerably similar, showing that there is a universal value for the indicators in iPTN. Our method offers a new choice to measure the importance and centrality of links.

In the aggregated international pesticide trade networks, all the indicators, that is, EB, hub, outdegree and outcloseness, conform to the formula $R_{\gamma }^\mathrm{{ascend}}>R_{\gamma }^\mathrm{{descend}}$. Among them, the difference corresponding to the EB indicator is the most obvious. Similar patterns were observed in the trade networks of the other five separate pesticide products.

**Figure 6:**
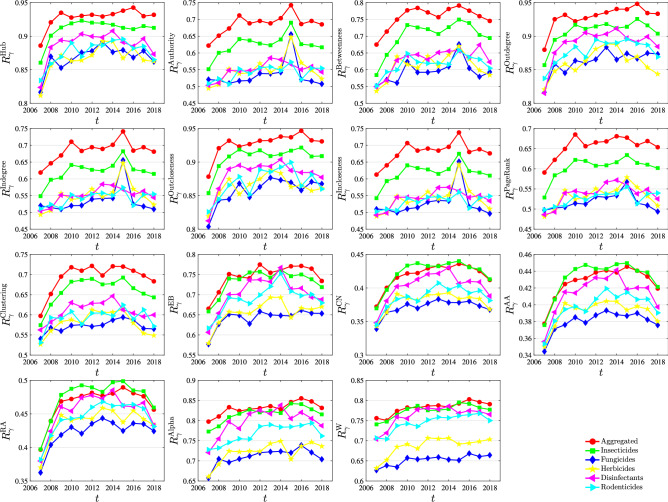
Comparison of the influence of different indicators on network robustness. In the legends, descend, ascend and random correspond to the cases of preferentially deleting the largest, smallest and random links of the indicator *I*, respectively. Each plot corresponds to an link importance or centrality indicator.

Figure 6 shows the evolution of network robustness under the 15 trade relationship importance indicators. Each plot corresponds to an indicator of relationship importance. Each line in each plot corresponds to a pesticide trade network. The comparison of different pesticide trade networks reveals that the robustness of the network under different strategies perturbations embodies a particular pattern; the aggregated network has the greatest stability, followed by insecticides (380891), disinfectants (380894), and rodenticides and other similar products (380899 ), herbicides (380893) and fungicides (380892). Of course, this pattern does not apply to all the indicators. The evolution of robustness of the pesticide trade network can be roughly divided into three stages: it was on the rise from 2007 to 2010; it was relatively stable from 2010 to 2015; it was on the decline after 2015. The 2007–2008 food crisis saw a sharp rise in global food prices, contributing to an international crisis and political instability, economic fluctuations and security deterioration in poor and developing countries. After the food crisis, the trade network is in the recovery period, and the trade relationship is restored and established. In 2015, the global pesticide market recorded the largest decline of over 10 years.

### Influence of vital links on the efficiency of international pesticide trade networks

**Figure 7:**
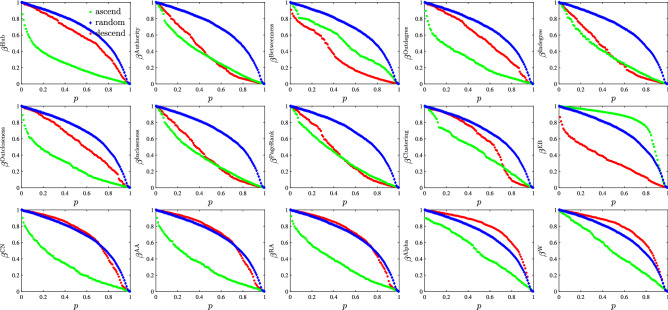
The relation between the proportion *p* of the deleted links and the network efficiency $\beta ^\mathrm{{I}}$ of the remaining network links after deleting the links according to the indicator *I*. In the legends, descend, ascend and random correspond to the cases of preferentially deleting the largest, smallest and random links with indicator *I*, respectively. Each plot corresponds to a link importance or centrality indicator.

As in the network robustness analysis, we use the same method to analyze the influence of vital links on network efficiency. Network efficiency measures the efficiency of matter or energy flowing in a network. We delete some vital links, recalculate the network efficiency $E_{p}$, and calculate its ratio to the original network efficiency *E*:

3 $$\begin{aligned} \beta ^\mathrm{{I}}(p)=\frac{E_{p}}{E}, \end{aligned}$$

where *I* represents the indicator and *p* is the proportion of deleted links. We delete links according to different sorting methods of the indicators. There are three methods for removing the trade relationships: “descend”, “ascend” and “random”. The result is shown in Fig. 7. The small area implies that this indicator plays an important role in the efficiency of the network structure, and the calculation formula is

4 $$\begin{aligned} R_{\beta }=\frac{1}{n+1}\sum _{k=0}^{n}\beta \left( p_k\right) , \end{aligned}$$

where $p_k=k/n$ represents the proportion of deleted links, and *n* represents the number of simulated deletions^57^.

In Fig. 7, the relationships between *p* and $\beta ^\mathrm{{I}}(p)$ embody different variation laws. Deleting the trade relationship with the biggest indicator *I* first (descend), the efficiency of the remaining network becomes smaller than that of the other two methods. In the case of deleting in ascending order of the indicators, the change of the network efficiency is the smallest. Those trade relations with the larger trade volumes have a greater effect on the efficiency of the network. This is mainly because the indicator of network efficiency is defined by the shortest path between nodes, and the shortest path is the weighted shortest path. Removing randomly the links of the trade network, we simulate 20 times, and then average the proportion $\beta ^\mathrm{{I}}$ of the efficiency in the case of “random”. In Fig. 7, when the proportion of deleted links with the biggest indicator reaches 20%, the proportion of efficiency in the rest of the network is approximately 20%.

**Figure 8:**
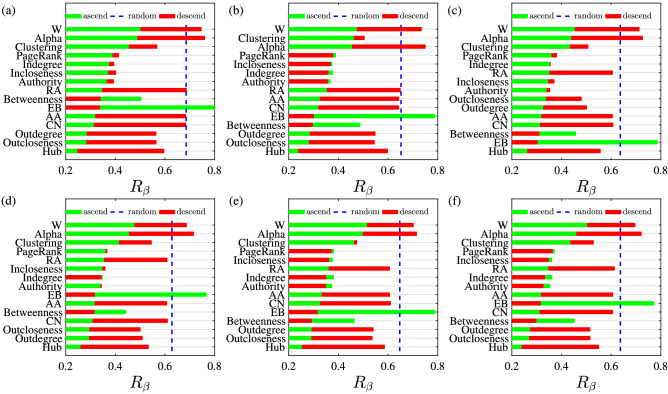
Comparison of the influence of different indicators on network efficiency. In the legends, descend, ascend and random correspond to the cases of preferentially deleting the largest, smallest and random links of the indicator *I*, respectively. The six plots correspond to the trade networks of pesticide products are (**a**) aggregated, (**b**) insecticides, (**c**) fungicides, (**d**) herbicides, (**e**) disinfectants, and (**f**) rodenticides and other similar products.

Figure 8 presents the comparison results of the influence of different indicators on network efficiency. Figure 8a is the combined network of five international trade networks for pesticide products and Fig. 8b–f corresponds to the trade networks of five pesticide products. The vital indicators based on the dual graph has significant effect, such as Auth, Indegree and Incloseness. These indicators are sometimes more effective than such traditional indicators as CN, AA and RA, defined by the original network trade relationship in Fig. 8b–f. The link betweenness (EB) of the original network is not as good as the centrality indicator defined based on the dual graph, either. The ranking of different indicators in the 6 networks is considerably similar in Fig. 8.

In Fig. 8, the indicators are sorted according to $\min {(R_{\beta }^\mathrm{{ascend}},R_{\beta }^\mathrm{{descend}})}$. There are more indicators that conform to the formula $R_{\beta }^\mathrm{{ascend}}>R_{\beta }^\mathrm{{descend}}$. Compared with the importance indicators based on original network, some link importance indicators based on dual graph have a far greater impact on network efficiency, and this finding also confirms the effectiveness of the link importance calculation method based on dual graphs proposed in this paper. However,our method are not meant to be applied to all cases of network function when identifying influential or important network links.

**Figure 9:**
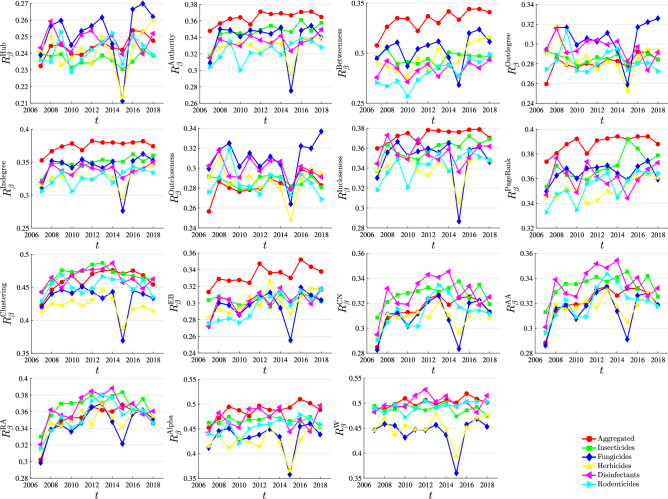
Comparison of the influence of different indicators on network efficiency. In the legends, descend, ascend and random correspond to the cases of preferentially deleting the largest, smallest and random links of the indicator *I*, respectively. Each plot corresponds to an link importance or centrality indicator.

Figure 9 shows the evolution of the network efficiency of trade relations under the impact of different strategies. As can be seen from the figure, there is a difference between network efficiency and network robustness. The values *R* of six types of networks are not the same. However, with the passage of time, the impact of network efficiency also had a period of rise from 2007 to 2008, then experienced a stable period, and finally had a period of decline. It is worth mentioning in the figure that there was a dramatic decline in the trade network in 2015, and then it rose rapidly. This phenomenon was observed under different indicators. The main reason is that the sales in the international pesticide market plunged 8.5% year on year, the steepest decline in more than a decade.

## Discussion

The comparison of the results of network efficiency and network robustness analysis shows that the indicators have different effects on different structural features. Therefore,different indicators can be considered for comparative analysis, and the optimal indicator, that is, the indicator with the greatest influence, can be deleted as the decision variable, which can more effectively specify a specific strategy. Our method of identifying vital links based on the dual graph can expand the set of candidate centrality indicators of links, so that more close comparisons can be made in the decision-making process, and more effective indicators can be found to measure the importance and centrality of network links.

In order to measure the importance of trade relations in trade networks, we adopt the idea of the dual graph in graph theory and convert the original pesticide trade network into a dual graph. The nodes in the dual graph correspond to the original trade relationship. The node centrality in the graph can be used as an indicator of the link centrality in the original network. We analyze the effect of different indicators on the stability of pesticide trade networks and compare them with some commonly used link centrality indicators. It is found that the nine indicators defined in the dual graph also have immense importance in the network stability and network efficiency.

The major innovation of this paper is that, based on the idea of dual graph in graph theory, the measurement of link centrality is transformed into the measurement of node centrality in dual graphs. All node-based analysis methods can be used in the analysis of network links through the corresponding dual graph. The contribution can be described in three aspects: The dual graph in graph theory is adopted, which provides new ideas and methods for analyzing networks; different indicators of network link importance and centrality are defined based on the dual graph; the relationship and effectiveness of the defined indicators and existing indicators are verified, and it is found that the indicators based on dual graphs perform better than some classic link centrality indicators in the original networks.

By studying the robustness evolution of the international pesticide trade network under the impact and disturbance of the trade relationship, it can be found that under some extreme events, the robustness of the trade network structure drops sharply, and then undergoes a recovery process. The numerical simulation and analysis of this paper can identify such changes in trade structure and provide guidance for constructing an optimal trade structure and a shock-resistant trade structure. In recent years, the robustness of the international pesticide trade network has declined, because trade wars and local wars have affected pesticide trade relations, and will have an impact on global food production and food security.

Since the case analysis concentrates on the international pesticide trade network, the conclusions drawn may not be applicable to other networks, such as the oil trade networks. Different network structures and different problem backgrounds determine the pros and cons of the measurement of link centrality. However, our method has good universality and scalability. It can be applied to different complex systems to analyze the importance of network relationships and the impact on the stability of the network structure and the network efficiency. Applying this method, we only need to convert the original network into a dual graph, which is characterized by better interpretability and provides a new perspective for network link analysis.

## Materials and methods

### Data

The data of this study is downloaded from the database of UN Comtrade, including all the export and import flows of pesticide products among more than 200 countries and regions in the world. The HS code of pesticides are 380891 (insecticides), 380892 (fungicides), 380893 (herbicides), 380894 (disinfectants) and 380899 (rodenticides and other similar products)^46^. This study uses the annual data from 2007 to 2018.

### Methodology

The most representative indicator of centrality for a link is link betweenness (EB), which measures the frequency of each link occurring on the shortest path between two nodes in the network^50,51^. In order to better measure the importance of different proportions of trade relations, we calculate the significance of each link based on the method of Serrano et al.^53^. We calculate the trade volume proportional matrix $W^{\mathrm {out}}$, where the element $w_{ij}^{\mathrm {out}}$ represents the ratio of economy *i* exported to economy *j*: $w_{ij}^{\mathrm {out}} = \frac{v_{ij}}{s_{i}^{\mathrm {out}}}$, where $s_{i}^{\mathrm {out}}$ is the total export of economy *i*. The import trade volume ratio matrix $W^{\mathrm {in}}$ can be similarly defined, where the element $w_{ij}^{\mathrm {in}}$ is expressed as $w_{ij}^{\mathrm {in}} =\frac{v_{ij}}{s_{i}^{\mathrm {in}}}$. $s_{i}^{\mathrm {in}}$ is the total import of economy *i*. The significance of each link $i\rightarrow {j}$ for node *i* and node *j* is defined as

5 $$\begin{aligned} \alpha _{ij}^{\mathrm {out}} = 1-(k_i^{\mathrm {out}}-1)\int _{0}^{w_{ij}^{\mathrm {out}}}\left( 1-x\right) ^{k_i^{\mathrm {out}}-2}dx, \end{aligned}$$

and

6 $$\begin{aligned} \alpha _{ij}^{\mathrm {in}} = 1-(k_j^{\mathrm {in}}-1)\int _{0}^{w_{ij}^{\mathrm {in}}}\left( 1-x\right) ^{k_j^{\mathrm {in}}-2}dx, \end{aligned}$$

where $k_i^{\mathrm {out}}$ is the out-degree of node *i*, and $k_i^{\mathrm {in}}$ is the in-degree of node *i*. The significance of each link $i\rightarrow {j}$ is defined as $\alpha _{ij}= \max \left( \alpha _{ij}^{\mathrm {out}},\alpha _{ij}^{\mathrm {in}}\right)$. It is represented by Alpha in this article. Significance $\alpha _{ij}$ is indicated only when the trade relationship is significant in the trade between the two parties. Generally speaking, the smaller the alpha is, the more important and significant the link is; the larger the other link importance indicators are, the more important the link is. considering the consistence with other link importance indicators, we add a negative sign before the alpha value here, which can be expressed as $\alpha _{ij}= -\max \left( \alpha _{ij}^{\mathrm {out}},\alpha _{ij}^{\mathrm {in}}\right)$.

The importance of trade relations can also be measured by the common neighbors of the two nodes corresponding to the link, which can be expressed as

7 $$\begin{aligned} \mathrm{{CN}}=\sum _{z\in \Gamma (i) \cap \Gamma (j)}\frac{w_{iz}+w_{zj}}{2}, \end{aligned}$$

where *z* is the set of common neighbors of nodes *i* and *j*, and $w_{iz}$ is the trade volume of economy *i* and neighbor *z*. In an unweighted network, it can be directly measured by the number of common neighbors; in a weighted network, it can be be expressed as the sum of the trade volume with common neighbors. However, if the common neighbor *z* has more neighbors or the trade volume is large, then the importance of the trade relationship *ij* is reduced. Hence, the Adamic/Adar ($\mathrm{{AA}}$) variable is introduced^55^

8 $$\begin{aligned} \mathrm{{AA}}=\sum _{z\in \Gamma (i)\cap \Gamma (j)} \frac{w_{iz}+w_{zj}}{\log (1+s_z)} \end{aligned}$$

where $s_z=\sum _{u\in \Gamma (z)}w(z,u)$. In order to increase the influence degree of neighbor’s trade volume, we calculate the Resource Allocation ($\mathrm{{RA}}$)^54^ as

9 $$\begin{aligned} \mathrm{{RA}}=\sum _{ z\in \Gamma \left( i\right) \cap \Gamma \left( j\right) }\frac{w_{iz}+w_{jz}}{s_z}. \end{aligned}$$

The three indicators CN, AA and RA have very similar properties and have a strong correlation.

#### Efficiency of iPTNs

In networks, we calculate the efficiency of network^48^ as

10 $$\begin{aligned} E=\frac{1}{N(N-1)}\sum _{i\ne j}\frac{1}{l_{ij}}, \end{aligned}$$

where $\frac{1}{l_{ij}}$ is the path efficiency between economy *i* and *j*, and $l_{ij}$ is the shortest path between the nodes *i* and *j* in the network. The greater the network efficiency, the stronger the connectivity between economies, and the greater the efficiency of energy flow and information flow.

#### Robustness of iPTNs

The indicators of robustness of network is defined as follows^56^:

11 $$\begin{aligned} R_{\zeta }=\frac{1}{n+1}\sum _{k=0}^{n}\zeta \left( p_k\right) , \end{aligned}$$

where $p_k=k/n$ represents the proportion of deleted links, and *n* represents the number of simulated deletions^57^. The larger the *R* value, the smaller the effect of removing the link on the network structure. The larger the indicator corresponding to the removal strategy, the smaller the impact of the trade relationship.

## Data Availability

Datasets related to this article can be found at https://comtrade.un.org/, an open-source online data repository.
